# Safety and tolerance of propranolol in neonates with severe infantile hemangiomas: a prospective study

**DOI:** 10.1038/s41598-017-01321-2

**Published:** 2017-05-04

**Authors:** Yi Ji, Siyuan Chen, Bo Xiang, Yang Yang, Liqing Qiu

**Affiliations:** 1 Division of Oncology, Department of Pediatric Surgery, West China Hospital of Sichuan University, Chengdu, 610041 China; 2 Pediatric Intensive Care Unit, Department of Critical Care Medicine, West China Hospital of Sichuan University, Chengdu, 610041 China

## Abstract

Although the efficacy of propranolol for the treatment of infantile hemangiomas (IHs) has been well documented, there is a paucity of clinical data regarding the safety and tolerance of propranolol in neonates. A prospective study of 51 patients less than 30 days of age with severe IH was conducted. All patients were admitted to the hospital for monitoring during initial propranolol treatment at day 0 with dose adjustments at days 7 and 28. Heart rate (HR), systolic blood pressure (SBP), diastolic blood pressure (DBP), blood glucose (BG) levels and potential side effects were evaluated during treatment. There were significant decreases in mean heart rate and SBP after the initiation of propranolol therapy (*P* < 0.05). In contrast, no significant differences in mean DBP and BG levels were observed after each dose during hospitalization (*P* > 0.05). Bradycardia and hypotension were noted in at least 1 recorded instance in 11.8% and 5.9% of patients, respectively. These hemodynamic changes were not persistent and were asymptomatic. Two patients who had a history of neonatal pneumonia reported severe bronchial hyperreactivity during treatment. This study demonstrated that propranolol administered to properly selected young infants was safe and well tolerated. However, close monitoring should be considered in high-risk young patients.

## Introduction

Infantile hemangiomas are the most common vascular tumor in children. If left untreated, the typical characteristic evolution of these tumors is rapid postnatal proliferation, stabilization and slow, spontaneous involution. IHs may be located on any region of the body but are mostly located on the skin of the head, face and neck. Although IHs are usually harmless, approximately 12–24% of IHs have complications. The most commonly occurring complication is ulceration, followed by visual compromise, airway obstruction, auditory canal obstruction and cardiac failure^1^. In many such cases, early intervention may be justified to potentially arrest the growth of the lesion, reduce associated complications, and avoid years of psychosocial concerns.

Previously, the standard treatment options for IH included laser, surgical excision or medical therapies such as corticosteroids. The options in life-threatening cases include treatment with vincristine, interferon-α or cyclophosphamide^1^. Unfortunately, none of these therapeutic modalities is ideal due to their restrictions or potentially serious side effects, such as temporary growth retardation, increased risk of infection and behavioral changes^2, 3^. In June 2008, Leaute-Labreze *et al*.^4^ reported their serendipitous discovery that oral propranolol was effective in the management of severe IHs. Subsequently, a growing number of studies further demonstrated that propranolol stops growth and induces an IH regression that is much better and safer than other treatments, including corticosteroids^5–9^. Currently, propranolol has been adopted as a first-line therapy for problematic IHs.

Regardless of subtype or depth, the largest increase in IH tumor size occurred at a mean age of 3 months, and by 5 months of age both segmental and localized IHs had reached 80% of their final size^10^. Remarkably, an elaborate study by Tollefson *et al*.^11^ further demonstrated that the most dramatic growth of IHs occurs between 5.5 and 7.5 weeks, which was much earlier than previously estimated. The authors of these studies suggested the need for a paradigm shift in the timing of referral and initiation of treatment for high-risk IH so that therapy can be initiated before or early during the most rapid growth, rather than after it is already completed. Although propranolol treatment can be efficacious beyond the proliferative phase, irreversible skin changes may have already occurred. In addition, recent studies have demonstrated that early treatment, especially when started during the proliferating phase, has been shown to be associated with better long-term outcomes^12, 13^. Therefore, for the IHs needing treatment, the ideal time to initiate therapy is either before or as soon as the evidence of permanent anatomic distortion or medical sequelae develop.

However, the use of propranolol in pediatric patients is not without risk. Known side effects of propranolol include hypotension, bradycardia, bronchospasm and hypoglycemia^14, 15^. Many clinicians remain cautious about the administration of oral propranolol for IHs, especially in young infants, because these drugs act systemically and affect the cardiovascular system. Although successful treatment with propranolol has been reported in premature or young infants, no detailed information on cardiovascular data and adverse events was provided^16, 17^. This prompted us to assess the safety and tolerance of propranolol in our young patients with the purpose of providing evidence-based data for future treatment recommendations.

The goal of this study was to assess the safety and tolerance of oral propranolol in young infants during treatment for severe IHs.

## Methods

A prospective study was performed in infants with problematic IHs who were hospitalized for propranolol initiation between October 2013 and September 2015. Approval was obtained from the Ethics Committee of West China Hospital of Sichuan University. All procedures followed the research protocols approved by Sichuan University and West China Hospital of Sichuan University and was conducted according to the Declaration of Helsinki. Parents gave written, informed consent for the use of propranolol in the treatment of IH.

This trial was registered on the public database ClinicalTrials.gov in January 2015 (NCT02342275). All patients were recruited in the Department of Pediatric Surgery, at West China Hospital of Sichuan University. Participation was offered to infants less than 1 month of age (corrected chronological age) with proliferating IH needing treatment that was defined as functional impairment, aesthetic disfigurement and whether IHs were ulcerated. The exclusion criteria were as follows: 1) patients who presented with contraindications to β-blocker therapy, such as allergy or hypersensitivity to propranolol, hypoglycemia, hypotension, second or third degree atrioventricular block, heart failure, severe bradycardia, bronchial asthma or bronchial obstruction; 2) patients with any acute illness or gastrointestinal diseases, especially those interfering with normal oral intake; and 3) patients who were unable to follow the assessment plan.

After initial evaluation, the patients’ parents who opted for oral propranolol therapy provided a thorough medical history (e.g., existence of comorbidities) and family history (e.g., cardiovascular disease). Physical examination, baseline electrocardiogram (ECG) and echocardiogram were performed in all infants. If cardiovascular abnormalities were detected, a pediatric cardiologist evaluated the patient to ensure that it was safe to initiate the propranolol treatment. Before treatment, the patients’ parents were provided a handout on safety monitoring during the treatment phase and were instructed to ensure that their children were free to feed as often they desired (at intervals not to exceed 4–6 hours).

All patients were scheduled for 24 hours of hospitalization for monitoring during initial propranolol treatment at day 0 with dose adjustments at days 7 and 28. Propranolol was initiated at a dosage of 1.0 mg/kg per day divided 3 times daily for 1 week (week 0), which was then increased to 2 mg/kg per day divided 3 times daily from day 7 (week 1). Patients were administered the first dose of propranolol at 8:00 am. To avoid the risks of hypoglycemia, we requested that propranolol be administered within 30 min after the patients were fed. During propranolol treatment, the doses were adjusted for weight gain (2 mg/kg per day).

Heart rate (HR), systolic blood pressure (SBP), diastolic blood pressure (DBP) and blood glucose (BG) values were the main outcome measures. Continuous bedside monitoring was performed on all infants during hospitalization by using a noninvasive multi-parameter monitor. HR, SBP and DBP were obtained before (baseline) and at 1, 2, 4 and 8 hours following the first dose of propranolol therapy (day 0). These values were also recorded before, and at 1, 2, 4 and 8 hours following the first dose of propranolol on week 1 (day 7) and week 4 (day 28) after dose escalation. Hypotension was defined as an SBP less than 50 mmHg and/or a DBP less than 30 mmHg^18, 19^. Bradycardia was defined as a HR less than 80 beats per minute (bpm) while awake or less than 60 bpm while asleep^20^. The BG level was measured by fingerstick using an automated glucometer, and obtained before (baseline) and 2 hours after the first dose of propranolol during hospitalization. BG level was also monitored before (baseline) and 2 hours after the first dose of propranolol on days 7 and 28. Hypoglycemia was defined as a BG level less than 40 mg/dL (2.2 mmol/L)^21^.

In addition to the analysis described, the frequency of adverse events (e.g., sleep disturbance, cool or mottled extremities, poor appetite, diarrhea, pneumonia) were reported by parents and collected by investigators during the follow-up examination (24 weeks).

Statistical analyses of the study were conducted using SPSS 19.0 for Windows (SPSS Inc., Chicago, IL). Data are expressed as the mean ± standard deviation (SD) for all paired statistical comparisons. Independent *t*-tests were used to analyze the quantitative data. *P* values less than 0.05 were considered significant.

## Results

### Patient demographics and hemangioma characteristics

**Figure 1:**
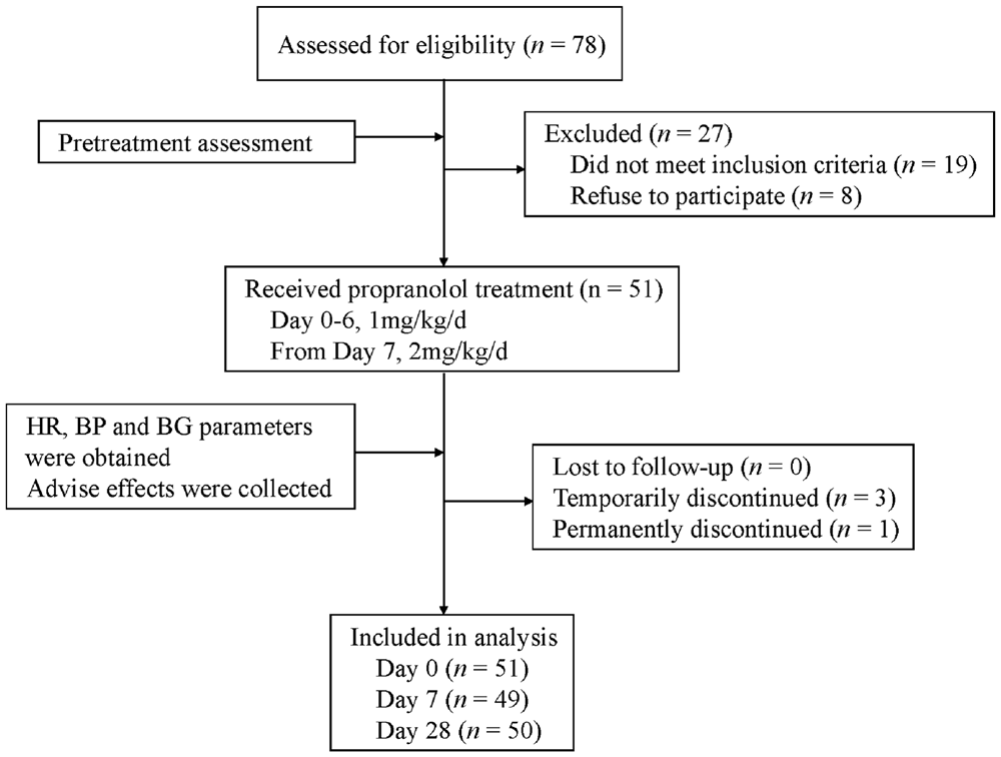
Study flowchart of the therapeutic regimen and monitoring protocol for propranolol treatment in young infants. HR, heart rate; BP, blood pressure; BG, blood glucose.

**Figure 2:**
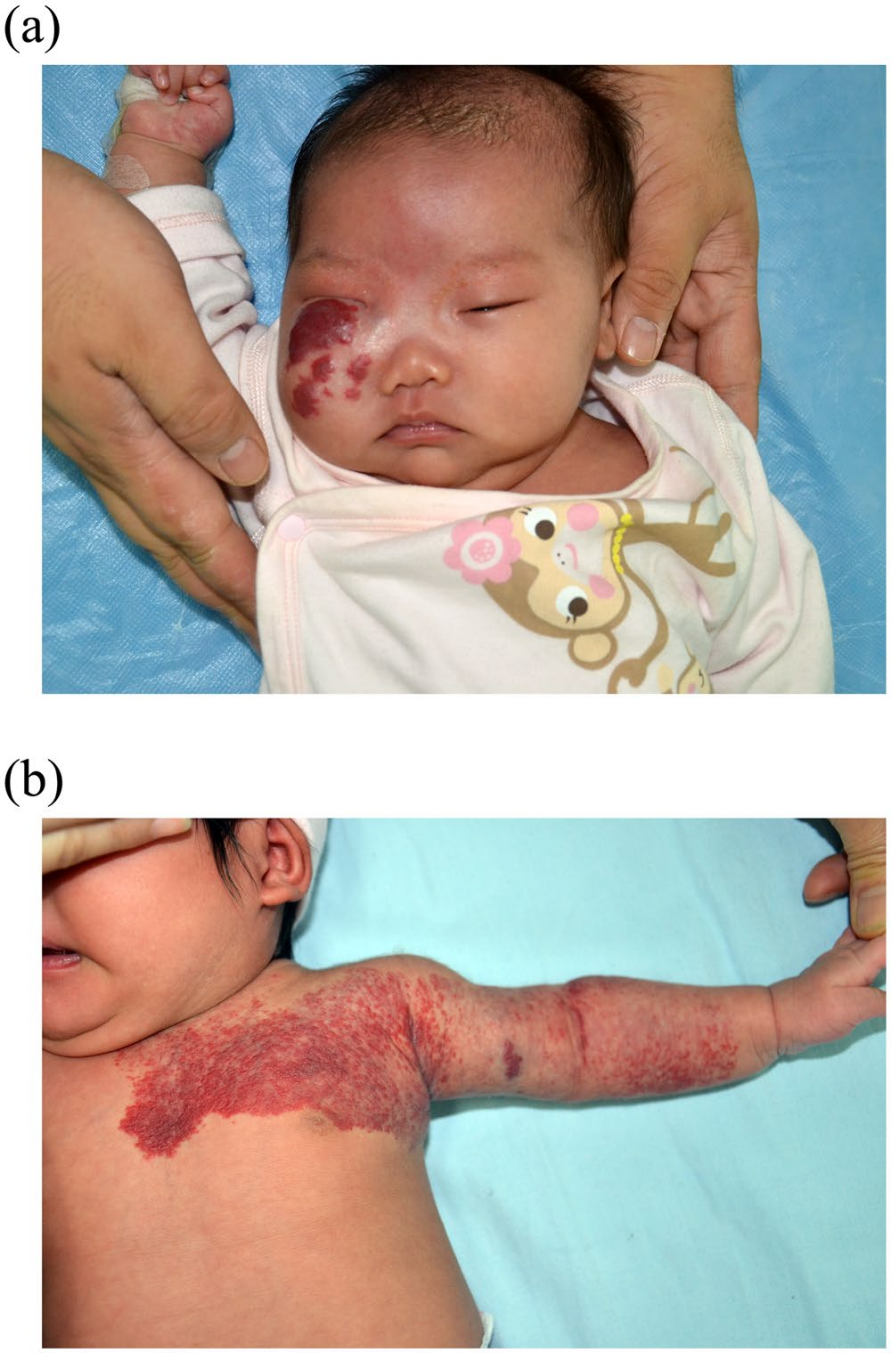
(**a**) The infantile hemangioma (IH) of this 26-day-old female affected the right cheek, eyelid and orbit, causing visual field cut. The lesion could proliferate, potentially resulting in permanent disfigurement and deprivation amblyopia. (**b**) The large, segmental IH of this 17-day-old female affected the left sternum, upper arm and forearm. This lesion had high a risk of ulceration and residual scarring.

Of 78 potential participants, 51 met the inclusion criteria and were hospitalized solely for propranolol initiation for the treatment of IHs (Fig. 1). Patient characteristics are summarized in Table 1. There were 14 males and 37 females, with a male-to-female ratio of 1:2.64. The median age at the start of propranolol therapy was 19 days (interquartile range, 15–24 days). Nineteen patients (37.3%) were premature infants. Three patients (5.9%) were treated with propranolol before reaching term equivalent age. The patients’ median weight at the time of propranolol initiation was 4.3 kg (range 3.8–4.9 kg). One patient weighted less than 2.5 kg at the time of propranolol initiation. Treatment indications for propranolol included: airway involvement and obstruction, vision compromise, bleeding and/or ulceration, feeding impairment, risk of permanent disfigurement or other high-risk IHs. The head-face-neck area was the dominant location (Fig. 2a). Among IHs in the cohort, 29.4% were of a segmental morphologic subtype (Fig. 2b).

**Table 1:** Baseline characteristics of patients and IHs.

Characteristic	Value
**Patients**	
Gender^†^	
Male	14 (27.5)
Female	37 (72.5)
Gestational age^†^	
Term born (≥37 weeks)	32 (62.7)
Born prematurely (<37 weeks)	19 (37.3)
Age at treatment (corrected chronological age, day)^‡^	19 (15–24)
Weight (kg)^‡^	4.3 (3.8–4.9)
ECG findings^†^	
Normal	48 (94.1)
Abnormal	3 (5.9)
Congenital heart defects^†^	
Yes	6 (11.8)
No	45 (88.2)
**IHs**	
Location^†^	
Head, face and neck	32 (62.7)
Extremity	10 (19.6)
Trunk	6 (11.8)
Liver	2 (3.9)
Subglottic area	1 (2.0)
Morphologic subtype^†^	
Localized	23 (45.1)
Segmental	15 (29.4)
Indeterminate	11 (21.6)
Multifocal	2 (3.9)

*ECG, electrocardiogram; IHs, infantile hemangioma. ^†^Values are presented as a number (percentage). ^‡^Values are presented as a median (interquartile range).

Four patients had a history of neonatal pneumonia. The pneumonia in these patients resolved before their discharge from the neonatal intensive care unit. Two patients had a parental history of cardiovascular disease, but neither of them showed any ECG or echocardiogram abnormalities. Three patients had abnormal ECG findings, including 2 with nonspecific intraventricular conduction delays and 1 with a right bundle branch block. Six patients had 1 or more congenital heart defects on echocardiography, including 3 with atrial septal defect, one each with ventricular septal defect, patent ductus arteriosus, mild coarctation of the aortic and mild pulmonary valve stenosis. The cardiologists allowed the administration of propranolol because these cardiac abnormalities are not absolute contraindications for the use of propranolol (Table 1).

### Heart rate

**Figure 3:**
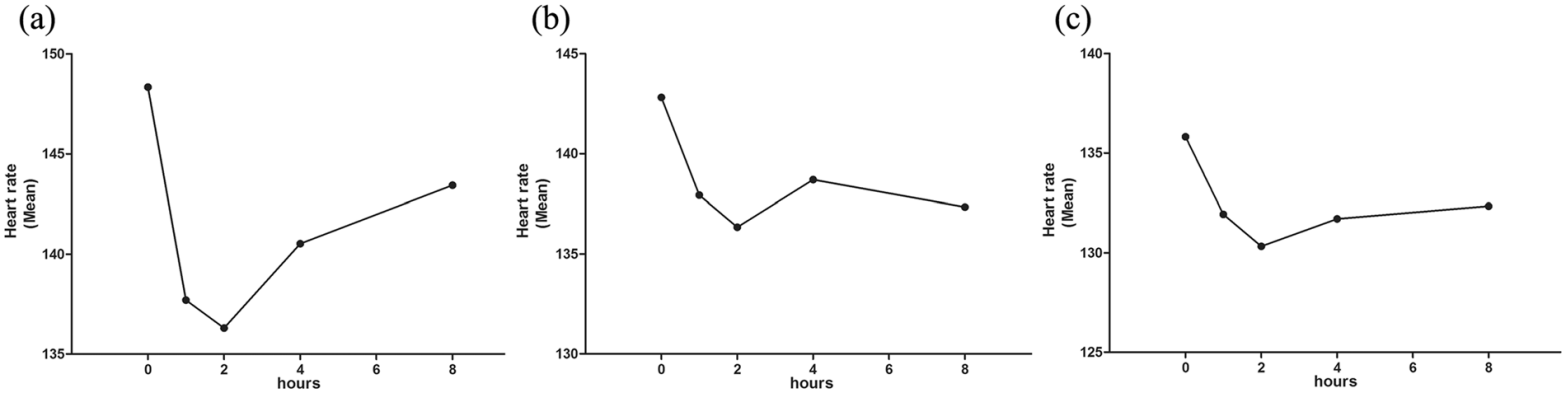
Changes in heart rate during oral propranolol treatment. Mean heart rate before and after the first dose of propranolol at weeks 0 (**a**), 1 (**b**) and 4 (**c**).

HR was carefully monitored during treatment. There was a dramatic decrease in HR after the initiation of propranolol therapy. Mean HR decreases occurred within 1 hour and were most apparent at hour 2 during the first 8 hours (Fig. 3a). Then, the HR gradually increased but was still found to be lower than the level prior to treatment. The mean levels of HR at hours 2 and 4 were statistically significant lower than baseline (*P* < 0.01 and *P* < 0.05, respectively). In contrast, there was no statistically significant difference in mean HR between baseline and at hours 8 (*P* > 0.05). All the decreases in mean HR were within normal limits, and all patients were clinically asymptomatic. HR fluctuations were also observed after the first dose on days 7 and 28. Interestingly, when patients were first administered propranolol at a dose of 2 mg/kg at week 1 (day 7), the HR decreased, but this decrease was less dramatic than was observed after the initiation of propranolol therapy on day 0. Similar results were obtained after the second dose adjustment at week 4 (day 28). HR fluctuations were less pronounced on days 7 and 28 than those observed on day 0 (Fig. 3b,c) (Table 2).

**Table 2:** Changes of mean heart rate during propranolol treatment.

Variable	Baseline	1 hour	2 hour	4 hour	8 hour
Day 0	148.3 ± 15.5	137.7 ± 14.3^†^	136.3 ± 16.7^†^	140.5 ± 15.0^*^	143.4 ± 14.6
Day 7	142.8 ± 15.2	138.0 ± 16.1	136.5 ± 16.3	138.7 ± 14.5	137.3 ± 13.8
Day 28	135.8 ± 14.4	131.9 ± 13.9	130.3 ± 14.5	131.7 ± 13.4	132.3 ± 14.2

*P < 0.05 when compared to the baseline values (0 hour); ^†^ *P* < 0.01 when compared to the baseline values. ^‡^Values are presented as a mean ± SD.

During the monitoring periods, bradycardia was noted in least 1 recorded instance in 11.8% of patients. All episodes of bradycardia were transient and were not associated with observable clinical symptoms.

### Blood pressure

**Figure 4:**
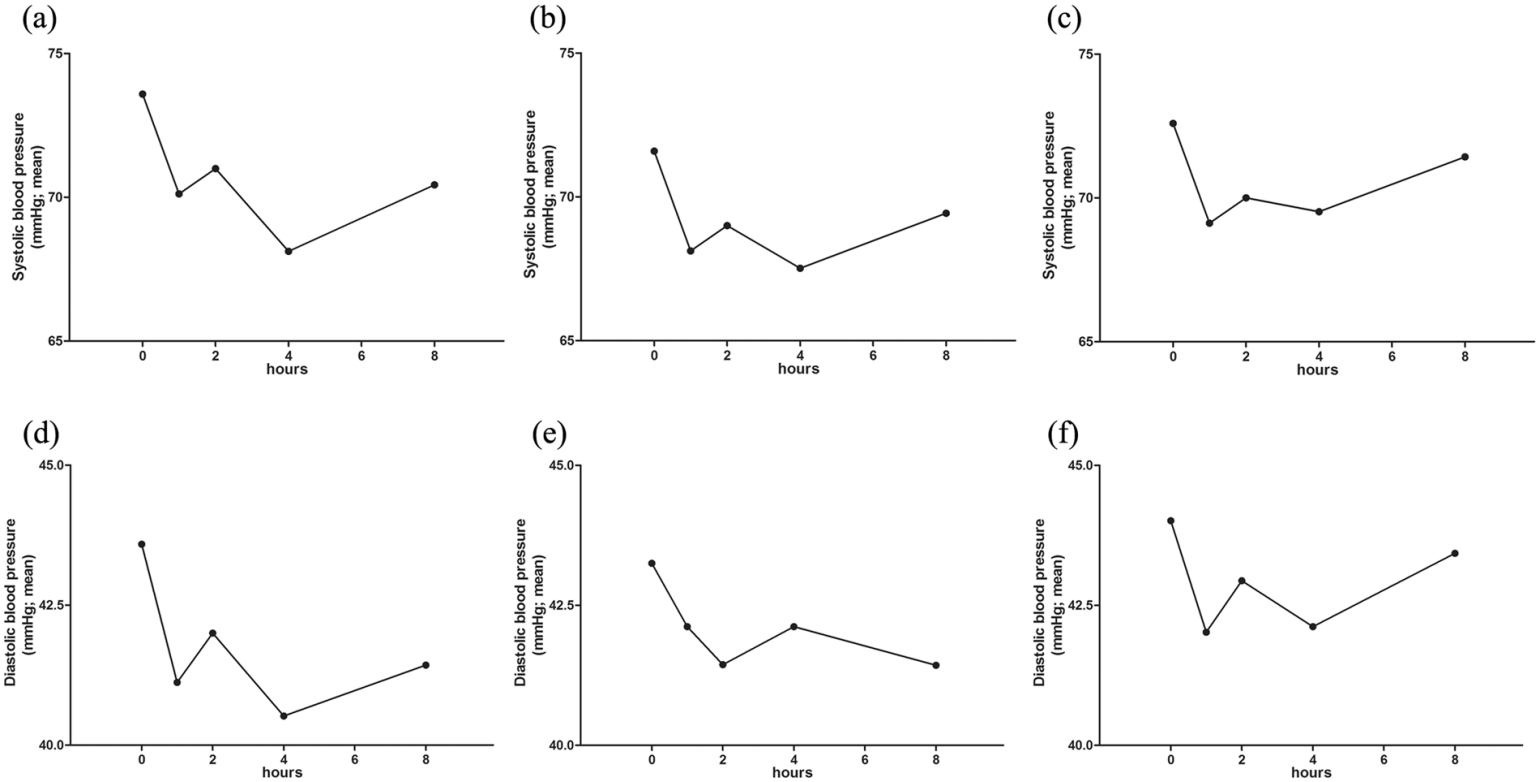
Changes in blood pressure during oral propranolol treatment. Mean systolic blood pressure before and after the first dose of propranolol at weeks 0 (**a**), 1 (**b**) and 4 (**c**). Mean diastolic blood pressure before and after the first dose of propranolol at weeks 0 (**d**), 1 (**e**) and 4 (**f**).

Like that observed with the HR, the decrease in mean SBP during the first dose of propranolol on day 0 was significant (Fig. 4a) (*P* < 0.05). In contrast, we did not observe a significant decrease in mean DBP after the initiation of therapy (Fig. 4d). Both SBP and DBP became more stable from the first dose on day 7 onward. Changes in mean SBP and DBP values on days 7 and 28 during dose escalation were not significant (Fig. 4b,c,e,f) (Table 3).

**Table 3:** Changes of mean systolic blood pressure (SBP) and mean diastolic blood pressure (DBP) during propranolol treatment.

Variable	Baseline	1 hour	2 hour	4 hour	8 hour
Day 0					
SBP	73.6 ± 11.2	70.1 ± 10.8	69.9 ± 12.0	68.1 ± 11.8^*^	70.4 ± 10.6
DBP	43.5 ± 10.9	41.1 ± 9.3	42.0 ± 10.4	40.5 ± 9.9	41.6 ± 11.5
Day 7					
SBP	71.7 ± 10.8	68.2 ± 9.9	68.9 ± 11.1	67.4 ± 12.2	69.2 ± 12.4
DBP	43.3 ± 9.5	42.6 ± 9.2	41.4 ± 9.7	42.8 ± 10.6	41.3 ± 10.1
Day 28					
SBP	72.5 ± 11.3	69.3 ± 11.9	70.5 ± 11.8	69.5 ± 10.8	71.5 ± 10.5
DBP	44.1 ± 9.0	42.2 ± 10.5	42.9 ± 9.3	42.4 ± 9.8	43.3 ± 8.8

*P < 0.05 when compared to the baseline values (0 hour). ^†^Values are presented as a mean ± SD.

Frequent deviations from normal blood pressure ranges occurred upon initiation of propranolol but patients were clinically asymptomatic. During the monitoring periods, hypotension was noted in least 1 recorded instance in 5.9% of patients. In all the patients, the identified hypotension was transient. No patient had a BP that remained <50/30 mmHg after further monitoring beyond the scheduled 8 hours. No patient was referred to a cardiologist based on results from the monitoring reports. None of the patients needed to discontinue the treatment or required dose modification due to hypotension.

### Blood glucose

**Figure 5:**
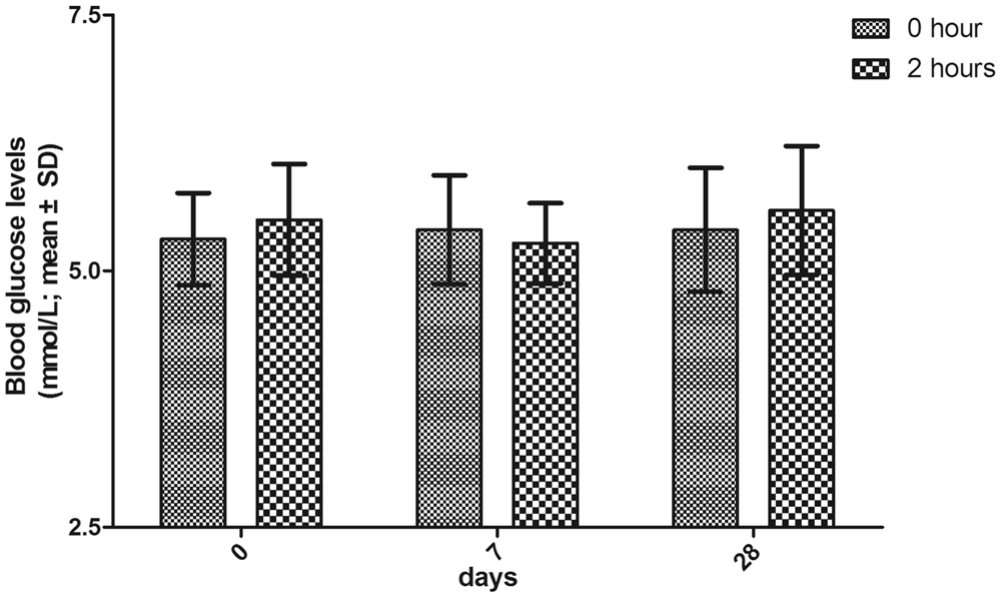
Changes in blood glucose level during oral propranolol treatment. Mean blood glucose levels before and after the first dose of propranolol at weeks 0 (**a**), 1 (**b**) and 4 (**c**).

BG values, carefully measured by paramedics, were normal in all patients. We did not see statistically significant changes in BP over the course of three days of monitoring (Fig. 5). No patient required a decrease in the dose of propranolol due to BG changes.

### Adverse effects and tolerance

All known adverse effects were recorded during the treatment of propranolol therapy. Thirteen patients developed diarrhea, and 1 patient required a rest period of 7 days prior to resuming treatment due to severe diarrhea. Eleven patients experienced sleep disturbances and 7 patients experienced bronchiolitis. Other common reported side events were agitation, cool extremities and vomiting, all of which subsided without discontinuing the medication (Table 4). Two patients who had a history of neonatal pneumonia reported bronchial hyperreactivity within 1 week after treatment initiation. The therapy was to be temporarily discontinued. However, one patient had severe respiratory symptoms again after resuming propranolol treatment. Therefore, oral propranolol was permanently discontinued in this patient.

**Table 4:** Adverse events with oral propranolol treatment to week 24 (*n* = 51).

Adverse events	n (%)
Diarrhea	13 (25.5)
Sleep disturbance	11 (21.6)
Bronchiolitis	7 (13.7)
Bradycardia	6 (11.8)
Vomiting	4 (7.8)
Cool extremities	4 (7.8)
Hypotension	3 (5.9)
Agitation	3 (5.9)
Bronchial hyperreactivity	2 (3.9)
Constipation	2 (3.9)
Viral upper respiratory tract infection	1 (2.0)

*Values are presented as number (percentage).

## Discussion

Most IHs do not cause serious morbidities and mortality in patients with IH is extremely rare. Therefore, the great majority of patients with IHs will not need treatment. Accordingly, the current perception assumes that most IHs should be left untreated. The treatment protocol of ‘watchful waiting’ (or initial observation) was considered and is still frequently encountered in clinical practice. Unfortunately, approximately 25–69% of patients with IH may develop a residual lesion after complete involution of the IH^22^. In addition, a significant minority of IHs can cause permanent disfigurement or functional compromise. Therefore, the risk-benefit analysis of any management must be scrutinized thoroughly, keeping in mind that a ‘watchful waiting’ strategy might often be appropriate, but in certain cases, timely intervention is crucial in minimizing long-term sequelae.

Young infants may have a greater likelihood of intolerance of hemodynamic changes than older children and adults. Clinically, there is controversy concerning the use of propranolol in young infants. Many studies did not include children who were younger than 30 days. In this study, we successfully provided clinical evidence of the safety and tolerability of propranolol in young infants with problematic IH. Previously, several large case series of patients treated with propranolol included infants younger than 30 days of age and/or premature infants, but no additional information on these patients was given. In this study, 37.3% of patients were premature infants. The median age of our patients at initial propranolol treatment was 19 days. Thus, the majority of our patients began treatment before or during the most rapid growth phase of IH. Remarkably, it has been demonstrated that facial IHs are 1.7 times more likely and segmental IHs are 11.0 times more likely to develop complications^23^. Therefore, the IHs in our cohort had a high risk for morbidity according to the anatomical location and/or the morphology of the hemangioma, which implied a great need for therapy.

Accurate measurement of HR and BP in neonates is sometimes difficult. The continuous bedside monitoring provided an accurate method for measuring HR and BP over long periods. This technique does not seriously interfere with normal behavior, and therefore, documentation of changes in HR and BP is possible during activities such as sleeping and is likely to provide more reliable information. In keeping with previous studies, we found that the effects of propranolol on HR in infants peaked approximately 2 hours after every oral dose^24^. Not surprisingly, and consistent with its use as an antihypertensive, during propranolol administration, there was a drop in blood pressure that persisted. Although the decreases in mean SBP were statistically significant, and 5.9% of our patients developed transient hypotension, all of the patients remained clinically asymptomatic. These data provided strong evidence that propranolol did not have a dramatic and persistent effect on hemodynamics in selected young infants.

Although rare, symptomatic hypoglycemia can be a serious complication of propranolol treatment. The exact mechanism or mechanisms by which propranolol exerts its influence on BG are not fully understood. It is conceivable that propranolol may block catecholamine-induced lipolysis, glycogenolysis and gluconeogenesis, which may facilitate hypoglycemia in children^25^. Young infants have lower glycogen stores. They are reliant upon their caregivers for their sustenance. In the present study, all of our infants were frequent feeders. Remarkably, our data demonstrated that there was no statistically significant decrease in BG with propranolol therapy in our patients. This finding is exciting and suggests that parental education on medication regimens and frequent feeding may be effective in preventing episodes of hypoglycemia in young infants.

Our data demonstrated that propranolol was generally well tolerated in young infants. However, it should be stressed that the satisfying results obtained in this study were based on the strict inclusion and exclusion criteria. Although some of our patients had associated cardiac abnormalities, none of them had contraindications for the use of propranolol. In addition, none of our patients had acute illnesses or conditions that would have interfered with normal oral intake. These strict inclusion criteria could largely reduce the possibility of hemodynamic risk in young infants.

On the other hand, recent studies have demonstrated that preterm infants with very low birth weight were more likely to be susceptible to the adverse effects of β-blockers^26, 27^. The authors of these studies suggested that β-blockers should always be cautiously administered in these patients. In addition, caution should also be used when treating young infants who had a history of apnea or bradycardia. In this study, the appearance of bronchial hyperreactivity in 2 patients was remarkable, suggesting that young infants (less than 30 days of age) who had a history of neonatal pneumonia appeared to be at higher risk for respiratory side effects. It is recognized that neonatal pneumonia can result in persistent alterations in lung function and airway responsiveness^28^. There is evidence that the potential risk of bronchial hyperreactivity was increased after infantile pneumonia^29^. For these potential high-risk infants, we propose brief inpatient hospitalization for monitoring during the induction of propranolol treatment or required dose modification.

## Conclusions

In conclusion, this study provided valuable data regarding the effects of propranolol on hemodynamics and BG levels in young infants. Our findings support the fact that oral propranolol administered on a progressive schedule was safe and well tolerated in properly selected young patients.
